# Investigating the rate of skeletal muscle atrophy in men and women in the intensive care unit: a prospective observational study

**DOI:** 10.1038/s41598-022-21052-3

**Published:** 2022-10-05

**Authors:** Ruo-Yan Wu, Wei-Hung Sung, Hui-Chen Cheng, Huan-Jui Yeh

**Affiliations:** 1 Department of Physical Medicine and Rehabilitation, Taoyuan General Hospital, Ministry of Health and Welfare, No.1492, Zhongshan Rd., Taoyuan Dist., Taoyuan, 330 Taiwan; 2 Department of Physical Therapy and Assistive Technology, National Yang Ming Chiao Tung University, Taipei, Taiwan; 3 Department of Ophthalmology, Taipei Veterans General Hospital, Taipei, Taiwan; 4 Department of Ophthalmology, School of Medicine, National Yang Ming Chiao Tung University, Taipei, Taiwan; 5 Program in Molecular Medicine, College of Life Sciences, National Yang Ming Chiao Tung University, Taipei, Taiwan; 6 Department of Life Sciences and Institute of Genome Sciences, College of Life Sciences, National Yang Ming Chiao Tung University, Taipei, Taiwan; 7 Brain Research Center, National Yang Ming Chiao Tung University, Taipei, Taiwan; 8 Institute of Public Health, National Yang Ming Chiao Tung University, Taipei, Taiwan; 9 Department of Physical Medicine and Rehabilitation, Taipei Hospital, Ministry of Health and Welfare, Taipei, Taiwan

**Keywords:** Epidemiology, Medical imaging, Risk factors

## Abstract

Muscle atrophy greatly affects the prognosis of patients in the intensive care unit, but the rate of change remains unclear. In this prospective observational study, we used ultrasound to measure the change in muscle thickness of the rectus femoris (RF) and vastus intermedius (VI) in 284 patients who were admitted to the SICU of Taoyuan General Hospital between January 1 and June 30, 2020. Patients were excluded if there is a wound at the right thigh which hinders the ultrasonography probe from placing. Daily rates of muscle atrophy were calculated using linear analysis and the ratios of change were plotted against the period of hospitalization. Patient characteristics were adjusted using propensity score matching and differences between men and women were analyzed. A linear mixed model was used to calculate the influence of other factors on muscle loss. The average daily atrophy rates of the RF and VI were 0.84% and 0.98%, respectively. The rate of atrophy was the highest in the third and fourth weeks. Daily atrophy rates of the RF and VI were approximately three times higher in women than in men. Protective factors of muscle atrophy included higher BMI and lower initial thickness of the RF and VI. Our study depicts the trend of muscle atrophy in the ICU and suggests more discussion in prevention to be conducted especially for women.

## Introduction

The incidence of intensive care unit-acquired weakness (ICUAW) is up to 80% in critically ill patients^1,2^. Due to its profound impact on the prognosis of patients, before or after hospital discharge^3,4^, developed countries have introduced early rehabilitation in the ICU to reduce the occurrence and severity of ICUAW^5,6^. At present, even with popularization of early rehabilitation, the rate of change in muscle mass with the number of days of hospitalization is not well-understood due to a lack of observational research in the field. Whether the rate of muscle atrophy trend is initially rapid and then gradual (i.e.: whether the slope is changed) due to improved postoperative inflammation or consistently rapid due to prolonged immobilization is unknown. This information can be acquired by observing daily muscle changes of patients under regular rehabilitation.

Physiological differences in the muscle cells of men and women affect the speed of recovery in both sexes. For example, women are more susceptible to disuse atrophy, but are better protected from inflammation-induced muscle atrophy (such as cancer cachexia)^7^. These differences may be due to the following reasons: (1) The proportion of type 1 muscle is higher in women^8^. (2) Women’s myofibrillar synthesis is faster than men’s when supplemented with nutrients^9^, although the muscle protein synthesis caused by intermittent exercise is worse than that of men^10^. (3) Men have more satellite cells and greater hypertrophy and proliferative capacity^11^. Studies that discuss sex differences in ICUAW are few^12,13^. Therefore, further research is required to determine the differences in the speed of muscle atrophy in men and women admitted in ICU.

Decrease in muscle strength is caused by polyneuropathy, myopathy, and/or muscle atrophy^14^. Moreover, several factors may have different effects on the speeds of atrophy of different muscle type. For example, muscle disuse can cause atrophy of type 1 muscle as they are sensitive to inactivity, decreased gravity, and denervation and tend to change from slow-twitch to fast-twitch muscles. In contrast, cachexia leads to preferential atrophy of type 2 muscle. Understanding these different rates of muscle atrophy of different types can help us better understand the pathogenesis of ICUAW^15,16^.

Considering the study requires multiple measurements and the ICU patients are mostly unstable, risks increase when there are more transferring to advanced neuroimaging, such as CT scan or MRI. Furthermore, acquiring samples of muscle by biopsy is invasive and causes discomfort, which may lead to a limited sample size. In comparison, ultrasonography is a convenient, non-invasive, valid measurement without radiation. We have chosen to investigate the RF and VI, which are mainly made of Type 2 and Type 1 muscle fibers^17^, respectively using ultrasonography. We designed a prospective observational study to determine the muscle atrophy curves of different muscle types in men and women who were admitted in the SICU.

## Methods

### Ethical considerations

This study was approved by the Taoyuan General Hospital Institutional Review Board (IRB number: TYGH108031) and all methods were carried out in accordance with relevant guidelines and regulations. The participants or their legal representatives provided written informed consent prior to the study. The consent of a legal representative was obtained only when the patient was unconscious.

### Design/setting/sample

This is a prospective observational study. Those were enrolled were patients admitted to the SICU of Taoyuan General Hospital between January to June 2020 and received routine and progressive rehabilitation 3–5 times per week. They had a variety of diseases affecting central nerve, respiratory and cardiovascular system. Patients who had a wound on the right thigh were excluded to avoid contact with the ultrasound probe. The observation time for each patient was from their first to the last day in the SICU or up to 28 days.

### Measurements

**Figure 1:**
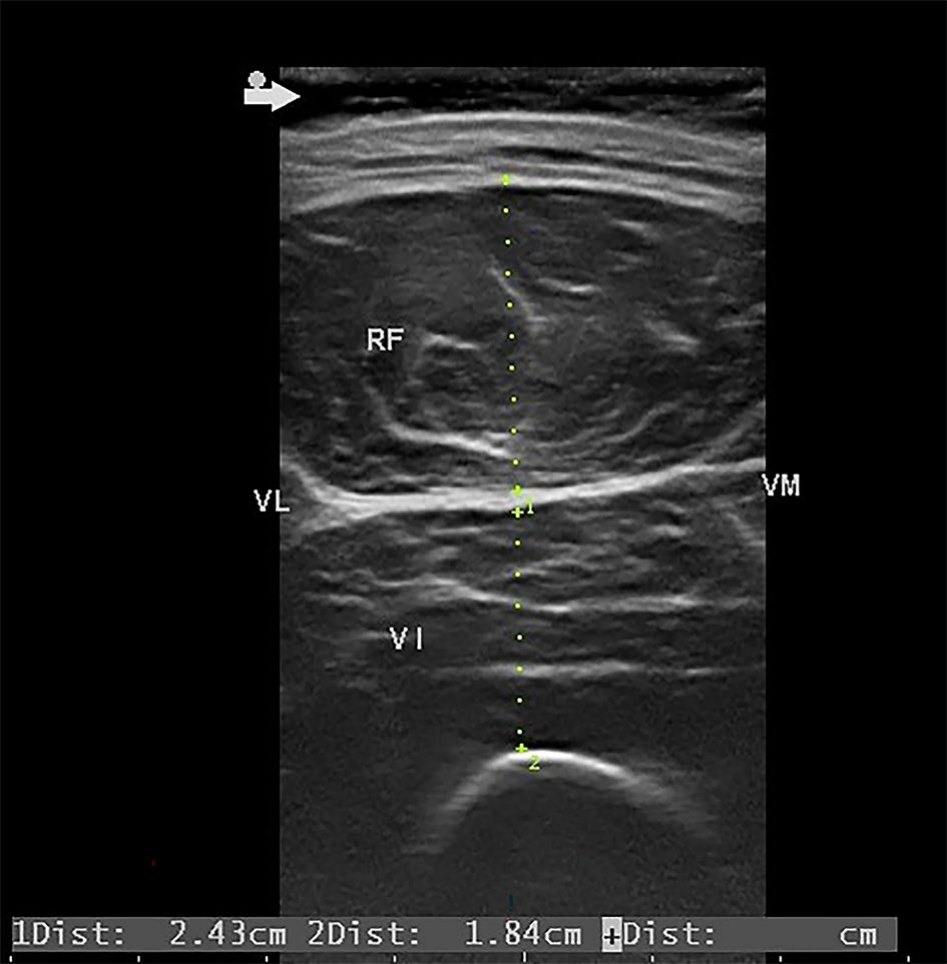
Ultrasound measurements RF: rectus femoris muscle, VI: vastus intermedius muscle, VL: vastus lateralis muscle, VM: vastus medialis muscle, Green dotted line 1: thickness of RF, Green dotted line 2: thickness of VI, Arrow: placement of the gel to ensure that there is no pressure on the muscles to produce deformation.

We used ultrasound^1^ (B-mode, linear probe, frequency of 8 MHz) to measure the thickness of the RF and VI. The patient was lying supine with their right foot extended. The ultrasound probe was placed in the middle of the anterior superior iliac spine and the upper edge of the patella on the right thigh^18^, perpendicular to the femoral bone. Figure 1 is an ultrasound image which shows the location of the RF and VI. To avoid changing the muscles’ diameters, additional pressure was not applied and the visible thickness of the conductive gel between the probe and the skin was maintained. One physician, who had more than 10 years of experience in skeletal muscle ultrasound, performed ultrasonography on all patients. The intra-rater reliability was 0.98, similar to that reported in other studies^19^.

Each patient underwent ultrasonography 5 days per week as each measurement was completed without data missing. The ratio of the muscle thickness measured on the day of examination to that measured on the first day was calculated for each patient. This ratio was considered a dependent variable.

Independent variables included number of days in the ICU, gender, age, height, weight, BMI, disease type, initial level of consciousness, and initial RF and VI muscle thicknesses. The disease types were categorized into central nervous system (CNS) diseases, including cerebral, subarachnoid, epidural, and subdural hemorrhage, brain tumor, and spinal cord injury; cardiovascular (CV) diseases, including rheumatic valve disease, endocarditis, coronary artery disease, and aortic dissection; thoracic diseases, including empyema, lung cancer, traumatic pneumo-hemothorax, and esophageal cancer; and infectious diseases, including empyema, limbs/intra-abdominal abscess, post-operation sepsis, and endocarditis. Disease classification was not mutually exclusive. Patients received Richmond Agitation-Sedation Scale (RASS) evaluation at hospitalization. We defined RASS of − 5 on the first day of admission as initial loss of conscious.

### Analysis

Each patient’s ratio of muscle thickness on each hospitalization day was calculated and the averages of all patients’ ratios were plotted against the number of days of hospitalization days to form a trend. Linear analysis was used to express the rate of muscle atrophy and calculate the slopes for the data of 4 weeks, the first 2 weeks, and the last 2 weeks.

Past studies have shown that factors, such as age, disease, and BMI may affect the proportion of muscle loss^20,21^. To adjust for confounding factors between men and women, we used 1:1 propensity score (PS) matching before performing the analysis^22,23^. PS for each patient was calculated based on age, diagnosis, height, weight, BMI, initial level of consciousness, initial RF and VI thickness, and admission days. The proportions of categorical variables and the mean ± standard deviation of continuous variables are shown in Table 1. The differences between the men and women groups were analyzed by Chi-square analysis for categorical variables and t-test for continuous variables. Finally, we used a linear mixed model to calculate the influence of each factor on the muscle loss and the significance was defined as p < 0.05. The software used was IBM SPSS Statistics for Windows, Version 23.0.

**Table 1:** The distribution of all observational characteristics of unmatched and propensity score-matched patients.

Characteristics		Before PS match			After PS match		
	All patients	Women	Men		Women	Men	
	Mean±SD	Mean±SD	Mean±SD	*P* value	Mean±SD	Ｍean±SD	*P* value
Admission (days)	18.28±20.0	17.62±16.9	18.57±21.2	0.154	18.8±17.7	21.5±23.4	0.089
Age (years)	62.6±17.6	67.6±16.9	60.5±17.4	<0.001	65.9±16.9	65.6±13.3	0.964
Height (cm)	162.4±8.8	164.2±8.8	161.7±8.7	<0.001	163.9±8.8	162.8±9.7	0.11
Weight (kg)	65.7±14.7	68.2±16.1	64.5±13.9	<0.001	69.6±15.8	68.0±17.0	0.191
BMI (kg/cm^2^)	24.9±4.7	25.7±4.7	24.5±4.6	<0.001	25.7±4.7	25.4±5.0	0.4
Initial RF (cm)	12.8±4.1	10.9±4.0	13.6±3.8	<0.001	11.4±3.9	11.7±3.0	0.177
Initial VI (cm)	13.4±5.7	11.0±5.4	14.5±5.5	<0.001	11.4±5.5	11.9±4.7	0.178

Disease type	All patients	Women	Men		Women	Men	
	Percentage	Percentage	Percentage	*P* value	Percentage	Percentage	*P* value
CNS	46.2%	44.9%	46.7%	0.526	46.6%	45.4%	0.762
Infection	13.6%	16.4%	12.4%	0.047	16.0%	17.4%	0.612
Thoracic	10.0%	7.8%	10.9%	0.079	8.0%	5.4%	0.174
CV	7.1%	7.4%	6.9%	0.774	8.9%	8.3%	0.787
Initial loss of conscious	40.0%	40.1%	39.9%	0.937	47.1%	41.7%	0.148

## Results

A total of 284 patients were enrolled from January 1, 2020, to June 30, 2020 during which no patient left the study. Among them, 195 were men and 89 were women. The distribution of characteristics of the unmatched and PS-matched patients is shown in Table 1. After the 1:1 PS match, there were no significant differences in any measurable confounding factors.

**Figure 2:**
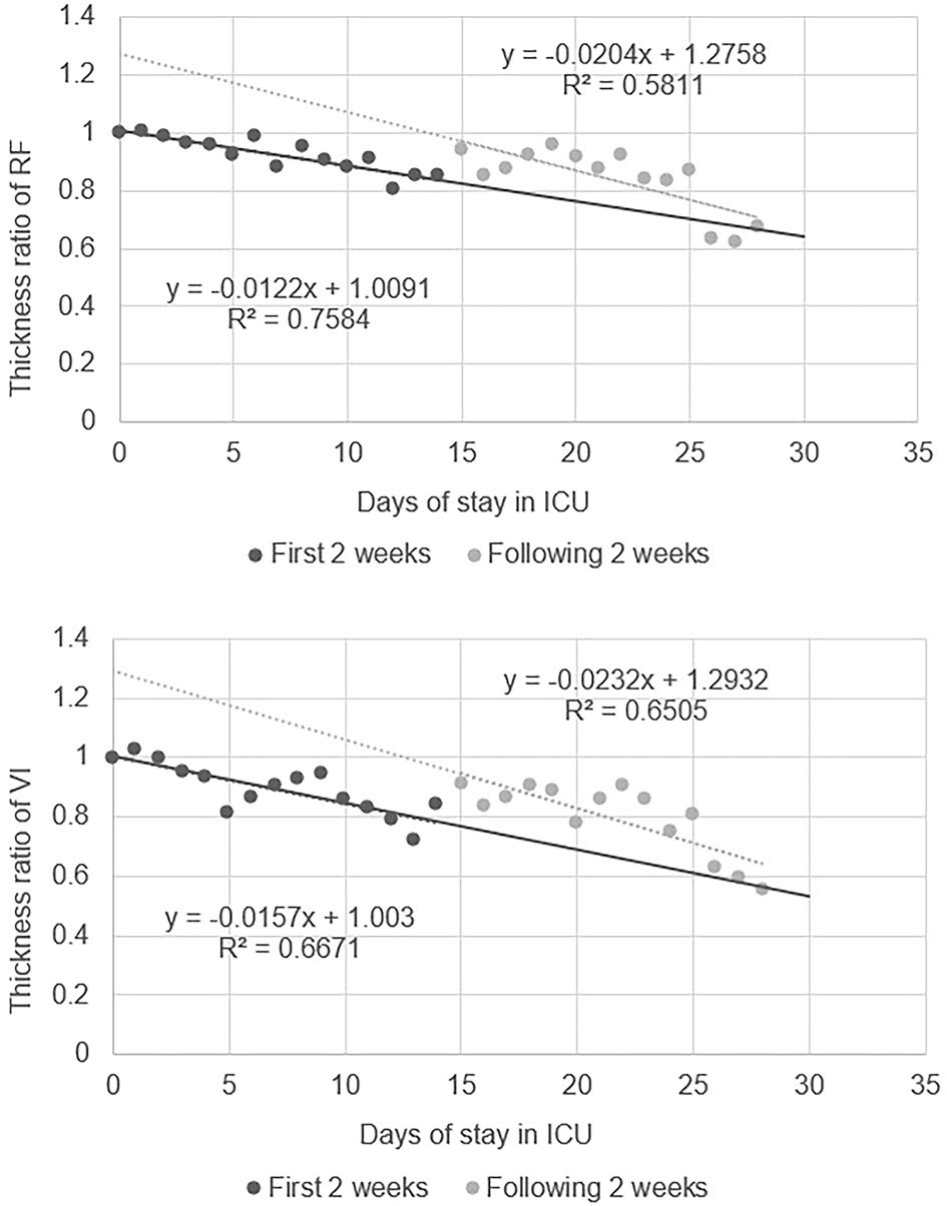
The regression line of daily change ratio of RF and VI in the first 2 weeks and the following 2 weeks.

We found that the proportion of the remaining RF and VI thicknesses decreased from 100% to 67.6% (SD = 21.6%) and 100% to 55.6% (SD = 23.4%), respectively within 4 weeks. The linear regression results showed that the daily atrophy rate of the RF was 0.84% and that of the VI was 0.98%. The rates of atrophy of the RF and VI in the first 2 weeks were 1.2% and 1.6%, respectively, while those in the second 2 weeks were 2.0% and 2.3%, respectively (Fig. 2). The rate of atrophy in the second 2 weeks was significantly higher than that in the first 2 weeks.

**Figure 3:**
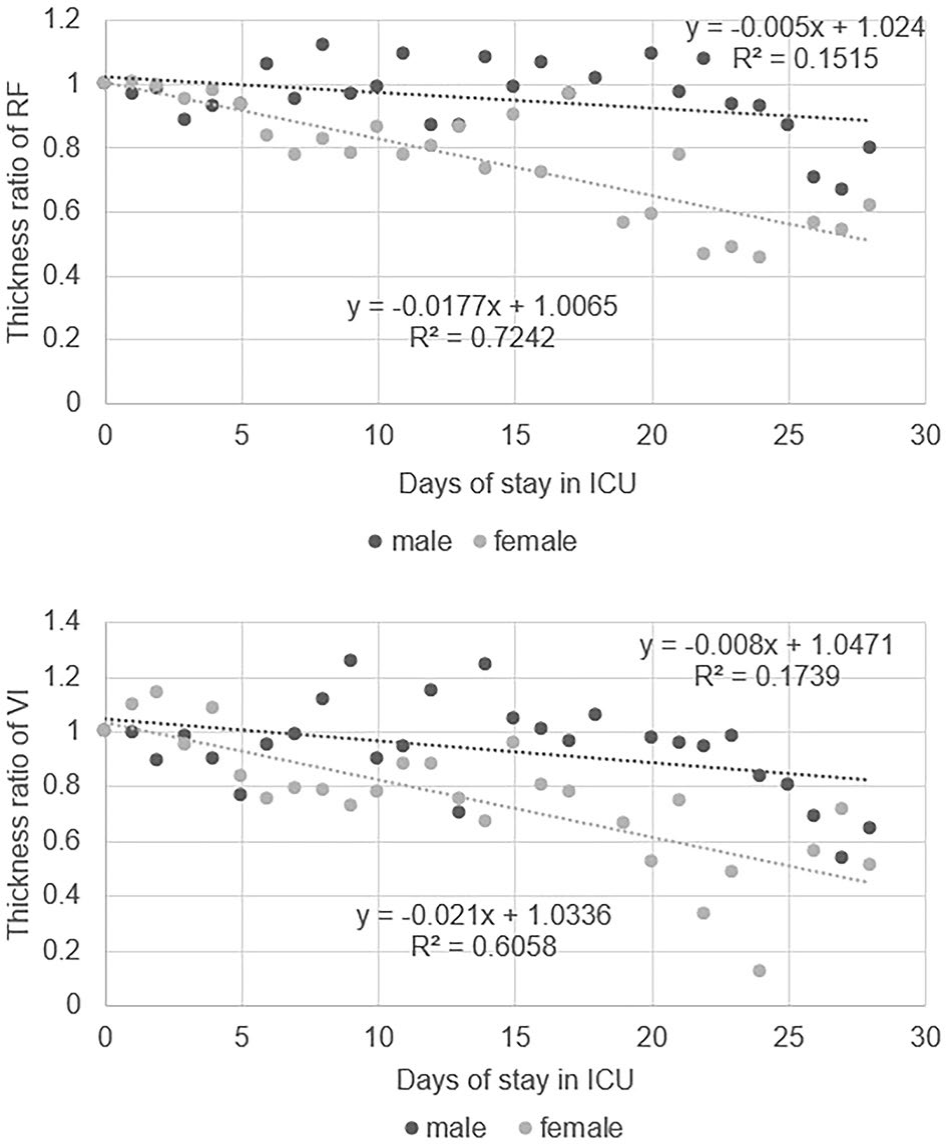
Daily ratio of changes in rates of atrophy of RF and VI in men and women.

The daily muscle thickness attenuation curve for men and women after PS-match is shown in Fig. 3**.** We found that the daily atrophy rate of women in RF is 1.7%, which is higher than that of men’s (0.5%). The daily atrophy rate of women in VI was 2.1%, which was higher than that of men (0.8%). Women’s muscle loss rate is approximately 2.6 to 3.4 times compared with that of men. (Non-PS-match result was shown in Supplementary Fig. S1)

By using a linear mixed model, we were able to calculate percentage change of muscle thickness attributed by each factor (eg. delta thickness divided by initial thickness). In women, the RF thickness will decrease by 8% compared to men. It will decrease by 0.6% with every admission day in the ICU and 0.5% with every 1-year increase in age. Further, RF thickness will increase by 0.7% with every 1-unit increment in BMI and decrease by 4% with every 1 mm increase in the initial RF thickness. There were no significant differences in the other factors (Table 2).

**Table 2:** The influence of each factor on RF/VI thickness using a linear mixed model.

Factors	The influence of each factor on RF		The influence of each factor on VI	
	Fixed effects estimates	95% CI	Fixed effects estimates	95% CI
Woman	− 0.080**	− 0.118 ~ − 0.042	− 0.057*	− 0.111 ~ − 0.003
CNS	0.010	− 0.037 ~ 0.056	− 0.019	− 0.085 ~ 0.047
Infection	− 0.039	− 0.096 ~ 0.019	− 0.148**	− 0.231 ~ − 0.066
Thoracic	− 0.062	− 0.142 ~ 0.018	− 0.032	− 0.146 ~ 0.082
CV	0.000	− 0.075 ~ 0.074	− 0.019	− 0.126 ~ 0.088
Initial loss of conscious	0.006	− 0.038 ~ 0.049	− 0.048	− 0.109 ~ 0.013
Admission days	− 0.006**	− 0.009 ~ − 0.003	− 0.007*	− 0.011 ~ − 0.002
Age	− 0.005**	− 0.006 ~ − 0.003	− 0.005**	− 0.006 ~ − 0.003
BMI	0.007**	0.003 ~ 0.011	0.007*	0.001 ~ 0.013
Initial RF/VI thickness#	− 0.040**	− 0.045 ~ − 0.034	− 0.032**	− 0.038 ~ − 0.027

*P value < 0.05, **P value < 0.001.

^#^ : “Initial RF thickness” used as a factor in RF analysis and “Initial VI thickness” used in VI analysis.

Similarly, with another linear mixed model, VI thickness in women will reduce by 5.7%. The VI thickness will be lost by 0.7% with every admission day in the ICU and 0.5% with every 1-year increase in age. Further, VI thickness will increase by 0.7% with every 1-unit increase in BMI and decrease by 3.2% for every 1 mm increase in initial VI thickness. In addition, VI thickness will reduce by 14.8% in patients with infectious diseases. There were no significant differences in the other factors (Table 2). The results of non-PS match data are given in Supplementary Table S1.

## Discussion

After evaluating the average daily muscle atrophy in association with the length of hospitalization, we found that the rate of muscle loss was the highest and most progressive in the 3rd and 4th weeks of the ICU stay. The VI, which is mainly constituted of slow-twitch muscles, atrophies slightly faster than the RF, which is mainly made of fast-twitch muscles. Moreover, we found that women are more susceptible to muscle loss than men, and the same results were observed for both type 1 and type 2 muscles.

Although many studies have explored the incidence and risk factors of muscle atrophy, there is a lack of discussion on the rate of muscle loss and its trend. Clair et al. and Kirby et al. described the changes in muscle loss in ICU patients over time^24^. However, in both studies, the sample sizes were 50 and 41, respectively, and were only observed over 7 days; hence, it was difficult to observe long-term trends in muscle loss. The sample size in the study by Puthucheary et al. was 63, but the observations were only carried out on the 1st, 3rd, 7th, and 10th day^25^. The study by Wolfgang et al. included 118 people and the study period was more than 28 days^26^. However, observations of each patient were only conducted twice, making it difficult to observe the daily changes in muscle thickness. Wolfgang et al. reported that patients' muscle loss was the most significant within 2–3 weeks of admission, after which the rate of atrophy plateaued. However, in our study, we found that the rate of muscle loss significantly increased in the 3rd and 4th weeks of admission. The results of our study suggest that more attention should be paid to preventing muscle loss for at least 1 month when managing patients in ICUs. Further studies with study periods longer than 1 month are required to explore long-term muscle thickness changes.

In this study, we used PS matching to exclude other interference factors and found that the muscle loss in women in the ICU was significantly higher than that in men, i.e., the decrease in rates of muscle loss of the RF and VI was 2.6 and 3.6 times higher, respectively. These results indicate that sex is an independent factor affecting muscle atrophy in the ICU, similar to the previous study conducted by De Jonghe B et al. in which Medical Research Council Scale was used to detect ICUAW in 2002^14^.

Similar to previous studies^16^, we did not find a significant difference between type 1 and type 2 muscle atrophies. Earlier studies have reported that disuse atrophy may be more obvious in type 1 muscles^15^; however, faster degeneration of the VI was not observed in patients with CNS injury or initial loss of conscious. This may be because many factors affect the patient's activity status. For example, in the ICU, restricted mobility in patients may result from CNS damage, general weakness, or sedation.

Suetta et al. reported that elderly patients lose muscle strength faster but lose muscle volume slower when compared to those in the young^27^. However, in our study, results showed that the older the person, the faster was the loss of muscle thickness. This may be due to the differences in BMI and other characteristics between older and younger patients. In the study by Suetta et al., the BMIs of the older patients were higher and the initial quadriceps muscle thicknesses were lower than those of the younger patients. These two factors were found to be protective factors for muscle atrophy in our study. We speculate that analyzing the differences between different age groups directly without adjusting for confounding factors is likely to yield confusing results. In contrast, in the study by Urso^28^, there was no significant difference in the initial muscle thickness between the elderly and the younger patients. Similar to our study, the results of the study by Urso showed that the rate of muscle atrophy was higher in the elderly compared to that of the younger patients.

Patients with higher muscle content than adipose tissue may be more likely to undergo muscle breakdown to meet the energy needs^29^. Our study showed that an increase in BMI is a protective factor for muscle atrophy, which implies that patients with higher proportions of adipose tissue have more energy resources to meet the body’s stress and recovery needs. This also suggests that we must suspect muscle atrophy more in lower BMI patients. However, in the study by Segaran et al., no difference was found in the degree of muscle loss between patients with higher and lower BMI^30^. This may be due to their small sample size and short observation time. Further studies are required to investigate the effect of BMI on muscle atrophy.

The limitations of this study are as follows: (1) Although past reviews have reported that medications, such as corticosteroids and neuromuscular blockers, were risk factors of ICUAW^2^, we did not analyze patients’ medication history in our study. As this is a longitudinal observational study, we cannot confirm the causal relationship between non-sustained medication status and daily changes in muscle ratios. Similarly, we did not consider laboratory data that can reveal possible changes in muscle breakdown, such as inflammation or infection, as independent variables. However, the lack of the abovementioned factors in our analysis is unlikely to affect the results as the aim of this study was to describe the differences in the rates of atrophy of different muscles between men and women. (2) The ICU setting in our study may not be similar to that in other countries. The mode and intensity of rehabilitation treatment and the attributes of patients may differ across different regions and countries. Considering the great variability of ICU admission, there are some factors (ex: fluid status, etiology) we overlooked which may affect the precision of measurement. (3) As we depicted the atrophy rate for as long as 4 weeks, it is likely the decline in the 3rd and 4th week was mostly attributed by, instead of all the patients in the ICU, those with more severe illness requiring longer admission days. (4) The observation time span (i.e. 6 months) is relatively arbitrary subject to funding and research time, although which is still longer than previous studies. (5) The number of men and women is disproportionate and beyond control of current study method. The effect of sex on muscle atrophy should be further verified with studies with higher level of evidence.

## Conclusions

In conclusion, the rate of atrophy of the RF in the first month in the ICU was 0.84% per day and that of VI was 0.98%, with the highest atrophy rates in the 3rd and 4th weeks compared to the first 2 weeks. The rate of muscle atrophy in women was approximately three times higher than that in men, which suggests that more attention must be paid to women in terms of prevention and treatment of muscle atrophy. Future studies should explore the factors and treatment effects of muscle atrophy in men and women separately.

## Supplementary Information

Supplementary Information 1.

Supplementary Information 2.

## Data Availability

The data that support the findings of this study are available on request from the corresponding author, HJY. The data are not publicly available due to their containing information that could compromise the privacy of research participants.

## Abbreviations

BMI: Body mass index
CNS: Central nervous system
CV: Cardiovascular
ICUAW: Intensive care unit-acquired weakness
RASS: Richmond agitation-sedation scale
RF: Rectus femoris
PS: Propensity score
SICU: Surgical intensive care unit
VI: Vastus intermedius
