# Supplementary material for: Investigating the rate of skeletal muscle atrophy in men and women in the intensive care unit: a prospective observational study
Source: Sci Rep. 2022 Oct 5;12:16629. doi: 10.1038/s41598-022-21052-3 (PMC9534861; doi:10.1038/s41598-022-21052-3)
Supplement: Supplementary file 1 — Supplementary Information 1. [file 41598_2022_21052_MOESM1_ESM.docx]

**Supplementary Figure 1** Daily ratio of changes in rates of atrophy of RF and VI in men and women without PS-match
