# Supplementary material for: Investigating the rate of skeletal muscle atrophy in men and women in the intensive care unit: a prospective observational study
Source: Sci Rep. 2022 Oct 5;12:16629. doi: 10.1038/s41598-022-21052-3 (PMC9534861; doi:10.1038/s41598-022-21052-3)
Supplement: Supplementary file 2 — Supplementary Information 2. [file 41598_2022_21052_MOESM2_ESM.docx]

**Supplementary-Table 1** The influence of each factor on RF/VI thickness using a linear mixed model without PS-match

| Factors | **The influence of each factor on RF** | | **The influence of each factor on VI** | |
| --- | --- | --- | --- | --- |
|  | Fixed effects estimates | 95% CI | Fixed effects estimates | 95% CI |
| Woman | -0.089** | -0.121~-0.057 | -0.047* | -0.090~-0.003 |
| CNS | 0.007 | -0.027~0.041 | -0.009 | -0.055~0.036 |
| Infection | -0.061* | -0.106~-0.016 | -0.174** | -0.236~-0.112 |
| Thoracic | -0.075* | -0.125~-0.025 | -0.074* | -0.142~-0.005 |
| CV | 0.009 | -0.047~0.064 | 0.004 | -0.072~0.080 |
| Initial loss of conscious | -0.019 | -0.050~0.013 | -0.051* | -0.094~-0.009 |
| Admission days | -0.008** | -0.011~-0.006 | -0.009** | -0.012~-0.006 |
| Age | -0.003** | -0.004~-0.002 | -0.002** | -0.003~-0.001 |
| BMI | 0.002 | -0.001~0.005 | 0.003 | -0.001~0.007 |
| Initial RF/VI thickness # | -0.032** | -0.036~-0.028 | -0.024** | -0.028~-0.021 |
| * : p value < 0.05, ** : p value < 0.001  # : “Initial RF thickness” used as a factor in RF analysis and “Initial VI thickness” used in VI analysis | | | | |
